# Temporal-frequency-phase feature classification using 3D-convolutional neural networks for motor imagery and movement

**DOI:** 10.3389/fnins.2023.1250991

**Published:** 2023-08-28

**Authors:** Chengcheng Fan, Banghua Yang, Xiaoou Li, Peng Zan

**Affiliations:** ^1^School of Mechatronic Engineering and Automation, School of Medicine, Research Center of Brain Computer Engineering, Shanghai University, Shanghai, China; ^2^School of Medical Instrument, Shanghai University of Medicine & Health Science, Shanghai, China; ^3^Engineering Research Center of Traditional Chinese Medicine Intelligent Rehabilitation, Ministry of Education, Shanghai, China

**Keywords:** brain-computer interface, electroencephalogram, motor imagery, convolutional neural networks, brain network analysis

## Abstract

Recently, convolutional neural networks (CNNs) have been widely applied in brain-computer interface (BCI) based on electroencephalogram (EEG) signals. Due to the subject-specific nature of EEG signal patterns and the multi-dimensionality of EEG features, it is necessary to employ appropriate feature representation methods to enhance the decoding accuracy of EEG. In this study, we proposed a method for representing EEG temporal, frequency, and phase features, aiming to preserve the multi-domain information of EEG signals. Specifically, we generated EEG temporal segments using a sliding window strategy. Then, temporal, frequency, and phase features were extracted from different temporal segments and stacked into 3D feature maps, namely temporal-frequency-phase features (TFPF). Furthermore, we designed a compact 3D-CNN model to extract these multi-domain features efficiently. Considering the inter-individual variability in EEG data, we conducted individual testing for each subject. The proposed model achieved an average accuracy of 89.86, 78.85, and 63.55% for 2-class, 3-class, and 4-class motor imagery (MI) classification tasks, respectively, on the PhysioNet dataset. On the GigaDB dataset, the average accuracy for 2-class MI classification was 91.91%. For the comparison between MI and real movement (ME) tasks, the average accuracy for the 2-class were 87.66 and 80.13% on the PhysioNet and GigaDB datasets, respectively. Overall, the method presented in this paper have obtained good results in MI/ME tasks and have a good application prospect in the development of BCI systems based on MI/ME.

## 1. Introduction

Motor imagery (MI) tasks in brain-computer interfaces (BCIs) have gained significant attention as a means to decode user intentions from brain signals, allowing individuals to control external devices through mental simulations (Pfurtscheller and Neuper, 2001; Aloise et al., 2010; Wolpaw, 2013). Electroencephalography (EEG)-based BCIs offer a non-invasive and portable approach with high temporal resolution, making them particularly suitable for MI applications (Nicolas-Alonso and Gomez-Gil, 2012). However, decoding EEG signals poses challenges due to high variability between sessions, subjects, and even within trials (Krauledat et al., 2008; Fazli et al., 2009). Additionally, the subject-dependent nature of time intervals, frequency ranges, and phase information further complicates the development of robust models applicable to a wide range of subjects. In this context, this paper aims to explore the potential of multi-domain dynamic feature extraction methods in MI-based BCIs and to enhance the accuracy and reliability of EEG-based MI control systems, opening new possibilities for improving the quality of life for individuals with disabilities or dyskinesia (Prasad et al., 2010; Wang et al., 2015; Zhang et al., 2016; Tariq et al., 2017).

In the process of decoding EEG signals in MI-BCI systems, effective feature extraction is particularly important. To the best of our knowledge, previous research has not adequately focused on preserving the temporal, frequency, and phase structures of diverse EEG features, which may limit the potential of the models. For instance, Park et al. (2014) and Kim et al. (2016) introduced the Strong Uncorrelating Transform Complex Common Spatial Pattern (SUT-CCSP) algorithm to handle EEG time-series data. Similarly, Handiru and Prasad (2016) proposed the Iterative Multi-Objective Optimization for Channel Selection (IMOCS) to choose the optimal combination of EEG channels. These studies only considered temporal information while overlooking the original spatial information. Brookes et al. (2016) combined connectivity measures with a multilayer network framework to capture connectivity features within and between frequency bands, but this approach neglected the temporal information while focusing on frequency information. Loboda et al. (2014) developed a large-scale synchronization method based on Phase-Locking Value (PLV) that leveraged phase synchrony between scalp-recorded activity in the sensorimotor and supplementary motor areas to compute differences between active and relaxed states. Similarly, Gu et al. (2020) calculated functional connectivity using PLV for α and β rhythm networks to investigate differences between left and right foot motor imagery, achieving a maximum accuracy of 75% and revealing the network mechanism of left and right foot MI. Leeuwis et al. (2021) aimed to study the relationship between EEG connectivity and users’ BCI performance using PLV as a measure of functional connectivity. With the similar measure of functional connectivity is Phase Lag Index (PLI), it can measure the asymmetry of the phase difference distribution between two signals and is robust to commonly used source signals. Both PLV and PLI are phase-related index to measure functional connectivity. By exploring brain functional connectivity through phase-based methods, valuable information flow between brain regions involved in MI can be thoroughly analyzed from the perspective of network structural features. However, these studies were limited to phase-based methods and overlooked the temporal and frequency information. Therefore, it is necessary to consider a novel feature extraction method that can integrate the diverse features of EEG signals, enabling the detection of more valuable and detailed motion-related information from EEG signals.

Recently, there has been widespread attention to deep learning (DL) methods for improving the classification performance of EEG tasks, which have been applied to EEG-based classification tasks. Lu et al. (2017) proposed an innovative DL scheme called the Frequential Deep Belief Network (FDBN) based on the restricted Boltzmann machine (RBM). This scheme utilizes fast Fourier transform (FFT) and wavelet packet decomposition (WPD) to obtain the frequency domain representation of EEG signals and trains three RBMs for motor imagery classification tasks. Dose et al. (2018) constructed an end-to-end model using convolutional neural networks (CNNs) for EEG signal classification and feature learning. Stieger et al. (2021) demonstrated that DL methods significantly improve offline performance in online MI-BCI and adapt to continuous control tasks compared to traditional methods. Additionally, detecting and utilizing neural biomarkers outside the motor cortex in full-scalp electrode montages can enhance performance (Zhang et al., 2022). Huang et al. (2022) proposed a novel model, the Local Reparameterization Trick-CNN (LRT-CNN), which combines local reparameterization and CNN to process raw EEG signals and achieved promising results in a four-classification task. Fan et al. (2023) presented a new algorithm that addresses the limitations of traditional 1D-CNNs in decoding motor imagery EEG signals by incorporating the Filter Band Combination (FBC) module and multi-view structure into the CNN. In summary, DL methods optimize the input attributes of EEG signals layer by layer and use various combinations of feature extraction expressions to obtain complex feature representations. This approach enables the acquisition of significant and distinctive features, thereby enhancing the analysis capability of EEG signals.

In this study, we proposed a method for generating Temporal-frequency-phase feature (TFPF) representation, which allows us to preserve the multivariate information of the EEG signal. To achieve this, instead of selecting a single time segment, we chose multiple time segments, which we referred to as a “time segments set.” After obtaining the time segments set, we constructed a multi-domain feature matrix (TFPF) for each time segment by applying bandpass filtering to the EEG signal. Considering the high variability within EEG recordings, in order to derive a robust model that can parameterize individual-dependent factors for a wide range of participants, we proposed a 3D voxel-shaped Temporal-frequency-phase dynamic feature representation by stacking multiple TFPF maps. Furthermore, recognizing the interdependencies between the three dimensions (Tran et al., 2015), we designed a compact 3D convolutional neural network (3D-CNN) model to learn the multivariate features from the 3D voxels.

In the context of deep learning, achieving high classification accuracy considering the available data is of utmost importance. However, gaining insights into the factors that contribute to individual differences in performance can provide a deeper understanding of cognitive processes and brain dynamics. Therefore, we captured the manifestation of multi-domain features in functional brain connectivity, including linear correlations, frequency-specific coherence, and phase synchronization. This comprehensive analysis enhanced our understanding of brain network dynamics and revealed individual differences in cognitive processes and brain activity within the EEG signals. Consequently, it improved the interpretability and level of understanding of EEG signals.

The main contribution of the proposed framework lies in the design of a compact 3D-CNN model, which significantly improves the decoding accuracy of MI/ME-based classification by preserving the multivariate information through the temporal-frequency-phase feature representation. Furthermore, the utilization of multiple features in brain network analysis overcomes the limitations associated with using a single feature type. This allows us to deepen our understanding of individual differences in cognitive processes and brain dynamics, as well as explore the inherent multi-faceted and complex network structures within EEG data.

## 2. Materials and methods

### 2.1. Description of dataset

The method proposed in this study has been employed to analyze two distinct datasets: the PhysioNet dataset^1^ (Goldberger et al., 2000) and the GigaDB dataset^2^ (Cho et al., 2017). These datasets are rich sources of EEG recordings capturing both motor imagery (MI) and real execution (ME) movements.

The PhysioNet dataset comprises EEG recordings from 109 subjects performing a range of motor tasks. EEG data were collected from 64 channels at a sampling rate of 160 samples per second, with each trial lasting 4 s. Subsets were created based on MI and ME movements of opening and closing left/right hands and both hands/feet, resulting in 2-class, 3-class, and 4-class classification tasks:

- **2-class**: MI left-hand vs. MI right-hand (**L vs. R**), MI both-hands vs. MI both-feet (**B vs. F**), MI left-hand vs. ME left-hand (**L vs. real L**), MI right-hand vs. ME right-hand (**R vs. real R**), MI both-hands vs. ME both-hands (**B vs. real B**), MI both-feet vs. ME both-feet (**F vs. real F**).
- **3-class**: MI left-hand vs. MI right-hand vs. MI both-hands (**L vs. R vs. B**).
- **4-class**: MI left-hand vs. MI right-hand vs. MI both-hands vs. MI both-feet (**L vs. R vs. B vs. F**).

The GigaDB dataset comprises EEG recordings obtained from a diverse group of 52 subjects (S1-S52) with a comprehensive coverage of 64 channels. The dataset offers a detailed examination of brain activity during MI and ME tasks for the left and right hand. Each task spans a duration of 3 s and the EEG data were sampled at a high frequency of 512 Hz.

### 2.2. Preprocessing of EEG signal

**Figure 1:**
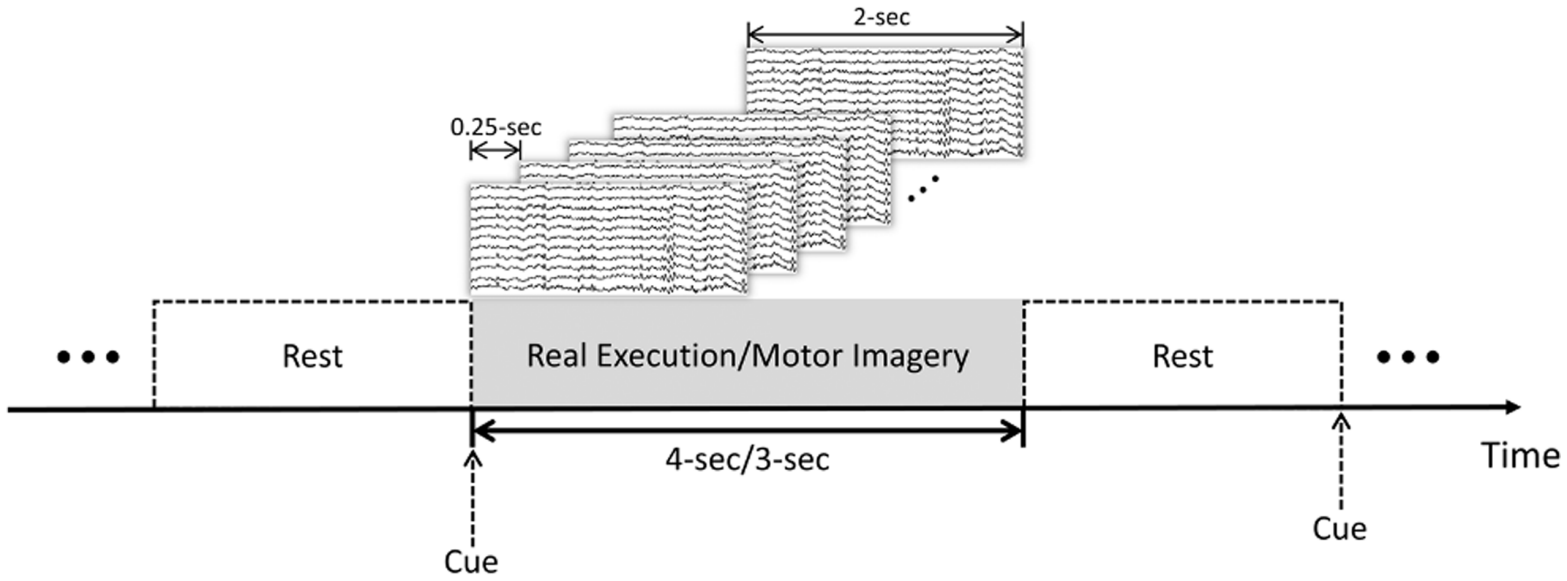
Trial-based sliding window segmentation: MI and ME tasks with rest intervals. 4-s for the PhysioNet dataset and 3-s for the GigaDB dataset.

In this study, 20 subjects from PhysioNet dataset and 20 subjects from the GigaDB dataset were selected to evaluate and validate the proposed method. Preprocessing steps, such as bandpass filtering within the range of 8–30 Hz and artifact removal using independent component analysis (ICA), were applied to ensure data quality. Due to the dynamic nature of the brain’s electrical activity, which can exhibit transient changes over time, and the potential challenges associated with maintaining sustained attention during the experiment, employing the sliding window method to divide the time series into smaller segments allows for a more effective capture and analysis of these dynamic patterns. This approach takes into account the temporal evolution of brain signals during MI and ME tasks, facilitating the extraction of crucial features and information necessary for accurate classification. Additionally, by focusing on shorter time periods, we can mitigate the potential influence of attention fluctuations or cognitive state changes that may occur over longer durations. To this end, we have chosen a sliding window step size of 0.25 s and window width of 2 s, as depicted in Figure 1. The division of the time series into multiple segments increases the diversity and richness of the training data, thereby enhancing the model’s ability to learn and generalize to unseen samples. The comprehensive evaluation and discussion of the impact of sliding windows on classification performance, encompassing aspects such as accuracy, stability, and generalization, will be thoroughly addressed in Section 3.1.1 of the paper.

### 2.3. Temporal-frequency-phase feature

Pearson’s correlation coefficient (PCC), Coherence (COH), and Phase-locking value (PLV) methods were used to extract EEG features in this study. PCC measures linear correlation, COH assesses frequency-specific correlation, and PLV quantifies phase synchronization. By combining these methods, we can capture temporal, frequency and phase dynamics of brain activity. This integrated approach provides a more comprehensive understanding of brain connectivity and improves the accuracy of EEG analysis.

#### 2.3.1. Pearson correlation coefficient

For the extraction of temporal features, PCC method was employed in this study. This approach measures the strength of linear relationship between two signals x(t) and y(t) , revealing their temporal connectivity. By calculating the correlation between signals, we obtained information regarding the direct connectivity among different brain regions. The PCC is defined as Niso et al. (2013):

(1) $$PCC=\frac{1}{N}\overset{N}{\underset{t=1}{\sum }}x\left(t\right)y\left(t\right)$$

The PCC ranges between −1 and 1. PCC values equal to −1 means complete linear inverse correlation between the two signals. PCC values equal to 0 means no linear interdependence. PCC values equal to 1 means complete linear direct correlation between the two signals.

#### 2.3.2. Coherence

For the extraction of temporal-frequency domain features, COH method was utilized in this study. Temporal-frequency coherence quantifies the extent of similarity or correlation between the frequency components of two signals. It provides a measure of the strength and consistency of the linear relationship between x(t) and y(t) across different frequencies f . It is obtained by squaring the module of the coherency function (K) . The coherency function is calculated as the ratio between the cross power spectral density, $S_{xy}\left(f\right)$ , of x(t) and y(t) , and their individual power spectral densities, $S_{xx}\left(f\right)$ and $S_{yy}\left(f\right)$ . By comparing the cross power spectral density of the signals to their individual power spectral densities, coherence allows us to assess the shared frequency content and coherence between the two signals. Thus, the coherence is defined as Niso et al. (2013):

(2) $$\begin{array}{l} K_{xy}\left(f\right)=\frac{S_{xy}\left(f\right)}{\sqrt{S_{xx}\left(f\right)S_{yy}\left(f\right)}}\\ COH_{xy}\left(f\right)={\left|K_{xy}\left(f\right)\right|}^{2}=\frac{{\left|S_{xy}\left(f\right)\right|}^{2}}{S_{xx}\left(f\right)S_{yy}\left(f\right)} \end{array}$$

The COH ranges between 0 and 1. COH values equal to 0 means no linear dependence between x(t) and y(t) at frequency f . PCC values equal to 1 means correspondence between x(t) and y(t) at frequency f .

#### 2.3.3. Phase locking value

For the extraction of phase features, PLV method was employed in this study. The PLV method measures the degree of synchronization between the phases of signals, reflecting the phase connectivity among brain regions. It is well-known that two coupled oscillators can exhibit synchronization even when their amplitudes are uncorrelated, which is referred to as phase synchronization (PS) (Rosenblum et al., 1996). Therefore, the following condition of phase-locking condition holds true for any given time t (Niso et al., 2013):

(3) $$\Delta \phi \left(t\right)=\left|\phi_{x}\left(t\right)-\phi_{y}\left(t\right)\right|\leq \mathrm{const}$$

Here, $\phi_{x}\left(t\right)$ and $\phi_{y}\left(t\right)$ represent the phases of the signals. However, in practical experimental systems, signals often contain noise and exhibit random phase slips of 2π. Therefore, it is necessary to address the cyclic nature of the relative phase using the following equation:

(4) $$\Delta \phi_{rel}\left(t\right)=\Delta \phi \left(t\right)\mathrm{mod}2\pi$$

The PLV is a measure of the inter-trial variability of phase difference at time t, ranging from 0 to 1. When there is little variability in the phase difference across trials (indicating strong phase synchronization), the PLV value approaches 1. Conversely, if there is significant variability, the PLV value approaches 0. The PLV is defined based on Eq. (4) as follows:

(5) $$\begin{array}{l} PLV=\left|\langle e^{i\Delta \phi_{rel}\left(t\right)}\rangle \right|=\left|\frac{1}{N}\overset{N}{\underset{n=1}{\sum }}e^{i\Delta \phi_{rel}\left(t_{n}\right)}\right|\\ \;\;\;\;\;\;\;\;\;\;\;\;\;\;=\sqrt{{\langle \cos\Delta \phi_{rel}\left(t\right)\rangle }^{2}+{\langle \sin\Delta \phi_{rel}\left(t\right)\rangle }^{2}} \end{array}$$

where 〈⋅〉 denotes time average.

### 2.4. Classification with 3D-CNN based on the TFPF

**Figure 2:**
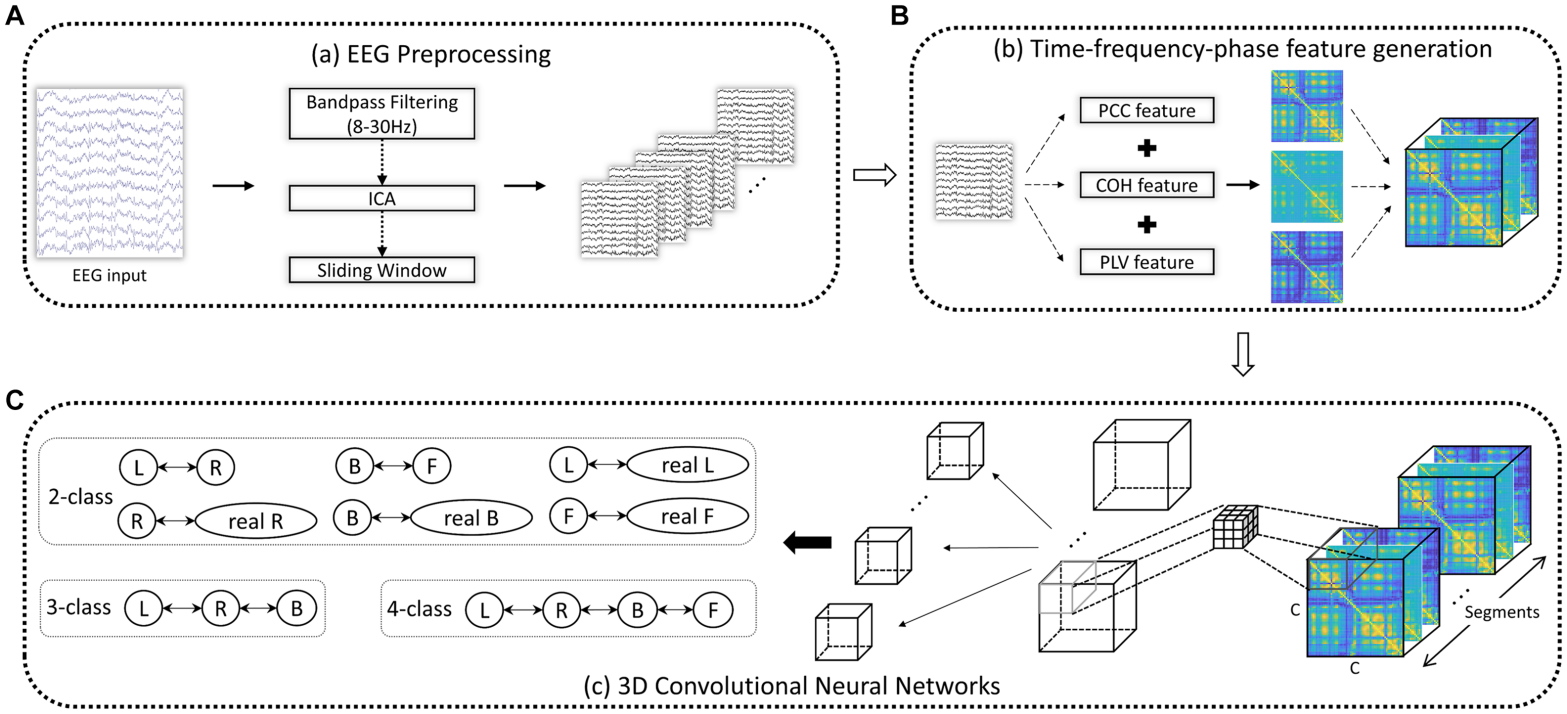
Schematic diagram of 3D-CNN based on the TFPF. **(A)** After EEG preprocessing, a series of EEG segments were obtained. **(B)** Given an EEG trial, a TFPF containing PCC, COH, and PLV was generated. **(C)** TFPF extraction using two-layer 3D-CNN. C: EEG channels.

To capture the intricate temporal, spectral, and phase dynamics of EEG signals, we employed sliding windows of varying lengths to collect the PCC, COH, and PLV features described in Section 2.3. This methodology allows us to derive comprehensive TFPF, providing a comprehensive representation of the EEG signals. The Schematic diagram of 3D-CNN based on the TFPF is shown in Figure 2.

#### 2.4.1. Temporal-frequency-phase feature generation

During the preprocessing stage, the information from the raw EEG signals was extracted through a series of steps. Firstly, bandpass filters were applied to the raw EEG signals to extract the EEG signals containing the alpha and beta frequency bands. Subsequently, the filtered signals were segmented using a sliding window strategy after the onset of the cue. Then, PCC, COH, and PLV were employed to obtain the temporal, frequency, and phase features of the signals.

**Figure 3:**
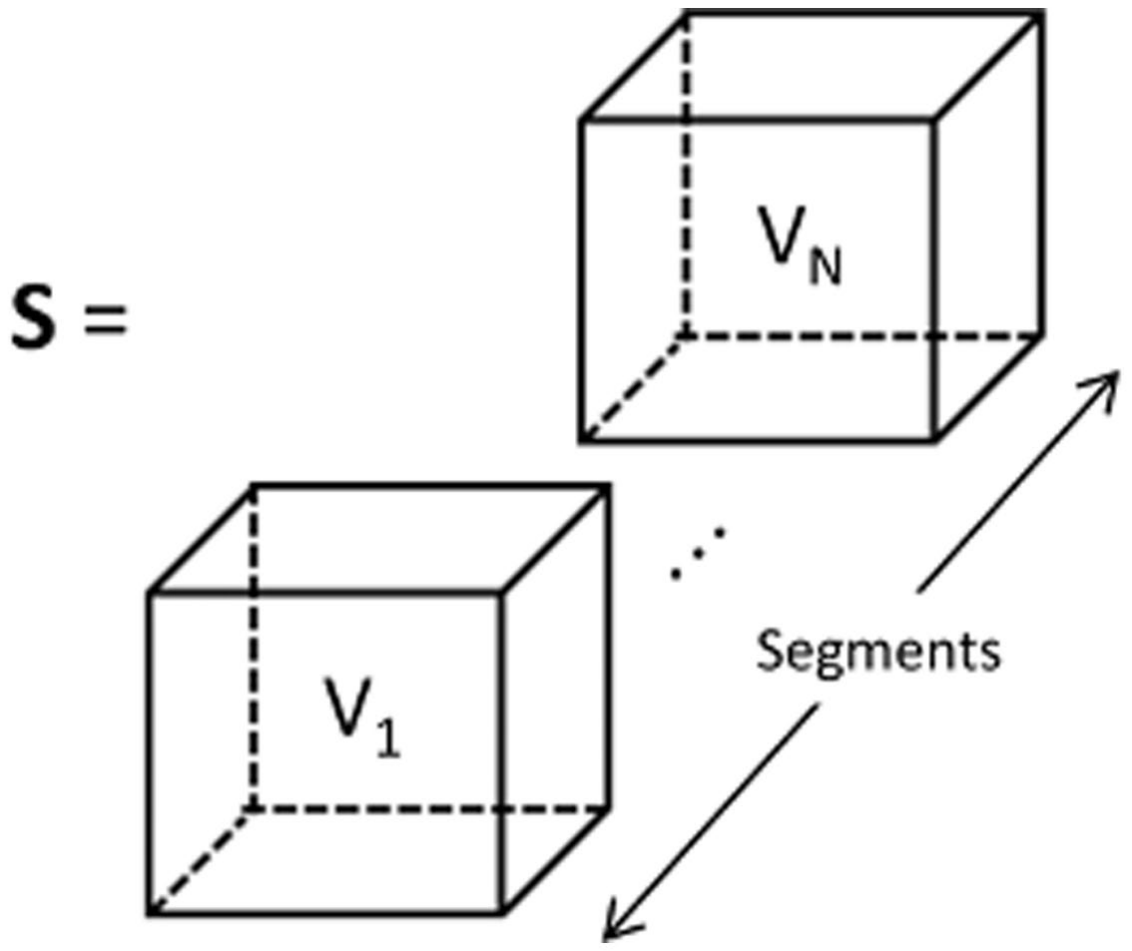
TFPF representation of input set which is denoted as **S**. Each voxel V_1_, …, V_N_, is obtained from EEG segments Seg_1_, …, Seg_N_.

Due to the inclusion of temporal, frequency, and phase information in the feature representation, it can be referred to as Temporal-Frequency-Phase Features (TFPF). Figure 3 illustrates the process of forming the feature representation. The input set (denoted as S) is constructed from TFPF representations of multiple EEG time segments. Each voxel (represented as V) in S corresponds to a TFPF representation generated from a segmented EEG signal. The detailed description of the feature representation generation method can be found in Algorithm 1. The algorithm sequentially describes the method for generating TFPF representations. Specifically, the EEG time segments were obtained from the preprocessing stage. Then, the PCC, COH, and PLV features were generated using the EEG time segment signals. Finally, the final feature representation was obtained by stacking the PCC, COH, and PLV features along the time segment axis.

By leveraging the complementary nature of PCC, COH, and PLV features, we can delve into various facets of brain connectivity and dynamics. This comprehensive approach not only allows for an effective characterization of EEG signals but also empowers us to extract crucial information pertaining to cognitive processes and neural mechanisms.
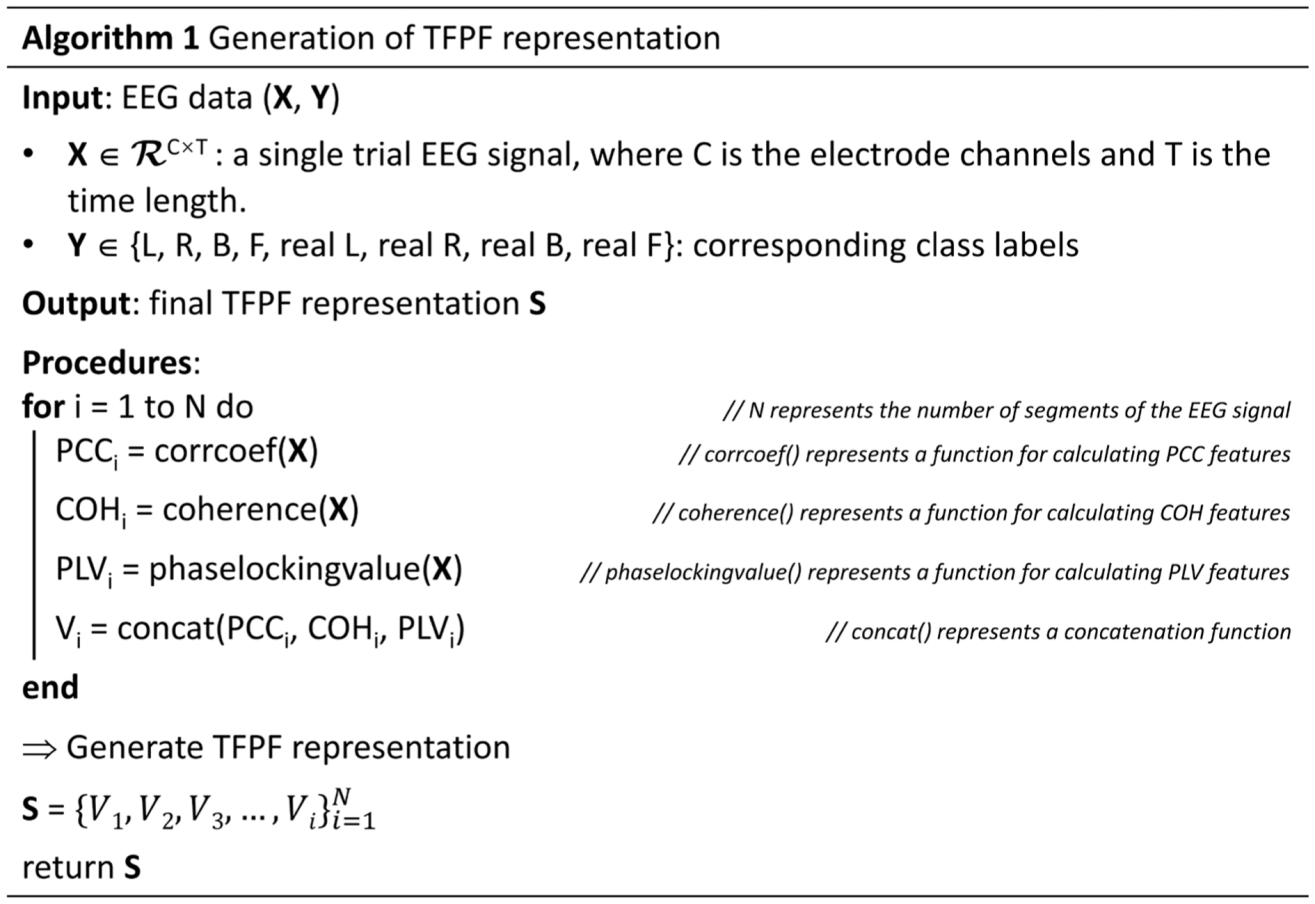


#### 2.4.2. 3D convolutional neural networks

For the extraction of multi-domain features encompassing the temporal, frequency, and phase domains, employing a 3D-CNN is a commendable approach. By leveraging three-dimensional convolution calculations, the 3D-CNN concurrently captures information from the temporal, frequency, and phase domains. This approach alleviates the cumbersome task of separate feature extraction and mitigates potential accuracy loss that may arise from employing distinct feature extraction functions. The integration of temporal, frequency, and phase domain information within the 3D-CNN framework allows for a comprehensive analysis of the intricate dynamics present in EEG signals. The synergy between the 3D-CNN architecture and the TFPF (Temporal-frequency-Phase Features) provides a robust and efficient framework for EEG signal classification, enabling the model to discern complex patterns and relationships among the different domains. In the context of EEG signal classification, we have observed that shallow networks tend to outperform deeper networks, unlike in image classification tasks. Recognizing this pattern, we have strategically crafted a streamlined network architecture comprising only two convolutional layers, as shown in Figure 2C. We configured the kernel size of the first convolutional layer to be 3 × 3 × 3, with a corresponding feature map dimension of 50. In the subsequent convolutional layer, the kernel size was determined by (C - 3 + 1) × (C - 3 + 1) × (N - 3 + 1), where C represents the input dimensions in one axis and N represents the input dimensions in another axis. The feature map dimension for this layer was set to 100. These carefully chosen settings ensure an effective extraction of features from the input data while increasing the network’s capacity to capture intricate patterns and relationships within the EEG signals. The 3D-CNN model employed ReLU activation functions during training, with each experiment consisting of 100 iterations. To optimize the cost function, we utilized the Adam optimizer, using a batch size of 16 and a learning rate of 1e-4. To assess the stability, accuracy, and reliability of the proposed model, we divided the data into training data (90%) and testing data (10%) and conducted experiments using 10-fold cross-validation. We adopted a 10-fold cross-validation technique to adjust and select different combinations of hyperparameters. Subsequently, we averaged the results from the ten experiments, ensuring the applicability of the testing data (test set) and demonstrating the reliability and robustness of the outcomes.

### 2.5. Brain network analysis

**Figure 4:**
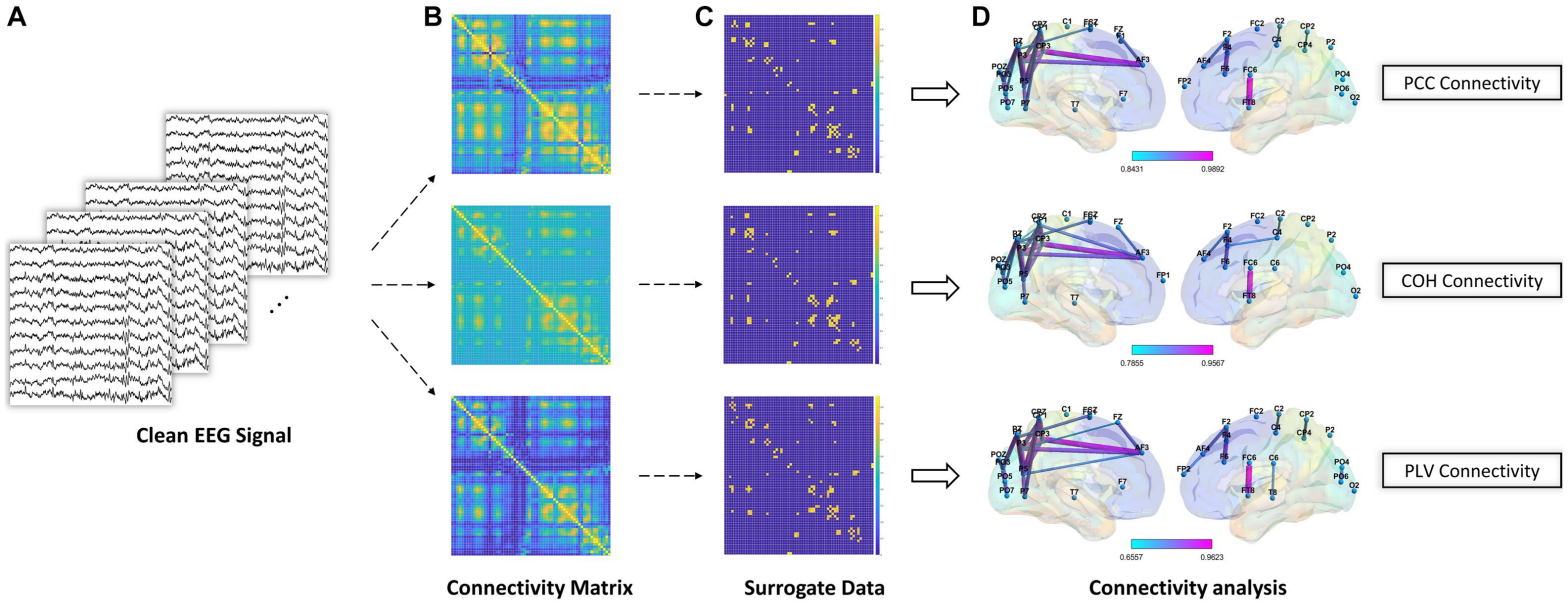
Schematic diagram of brain network analysis. **(A)** The clean EEG signal was obtained during EEG preprocessing. **(B)** PCC, COH, and PLV feature representation for connectivity analysis. **(C)** Surrogate data method to remove spurious connections. **(D)** Brain connectivity analysis using significant connections after using surrogate data method.

When it comes to brain network analysis, we delve into the intricate connections and information flow within the brain. By studying brain networks, we uncovered the interactions between different brain regions, the pathways of information transmission, and the coordination of brain functions, aiding in our understanding of the fundamental mechanisms underlying brain function and cognitive processes. As shown in Figure 4, After the EEG preprocessing steps, we computed the connectivity matrix using clean EEG signal (i.e., the data processed by removing artifacts, bandpass filtering, and ICA). Subsequently, we employed surrogate data analysis to remove spurious connections in the connectivity matrix. The underlying principle of surrogate data analysis involves independent and random shuffling of the phase of the Fourier coefficients of the time series, resulting in the generation of surrogate time series (Li et al., 2016). In this study, for each subject and each trial, we performed 200 random shuffles to generate surrogate time series for each edge.

## 3. Experiment and results

In this section, the proposed method was first used to classify the TFPF of EEG signals, which included ablative experiment and subject-independent classification. Then, brain network analysis was performed to analyze the brain network topology and its impact on the experimental results.

### 3.1. Temporal-frequency-phase feature classification

#### 3.1.1. Ablative experiment

**Figure 5:**
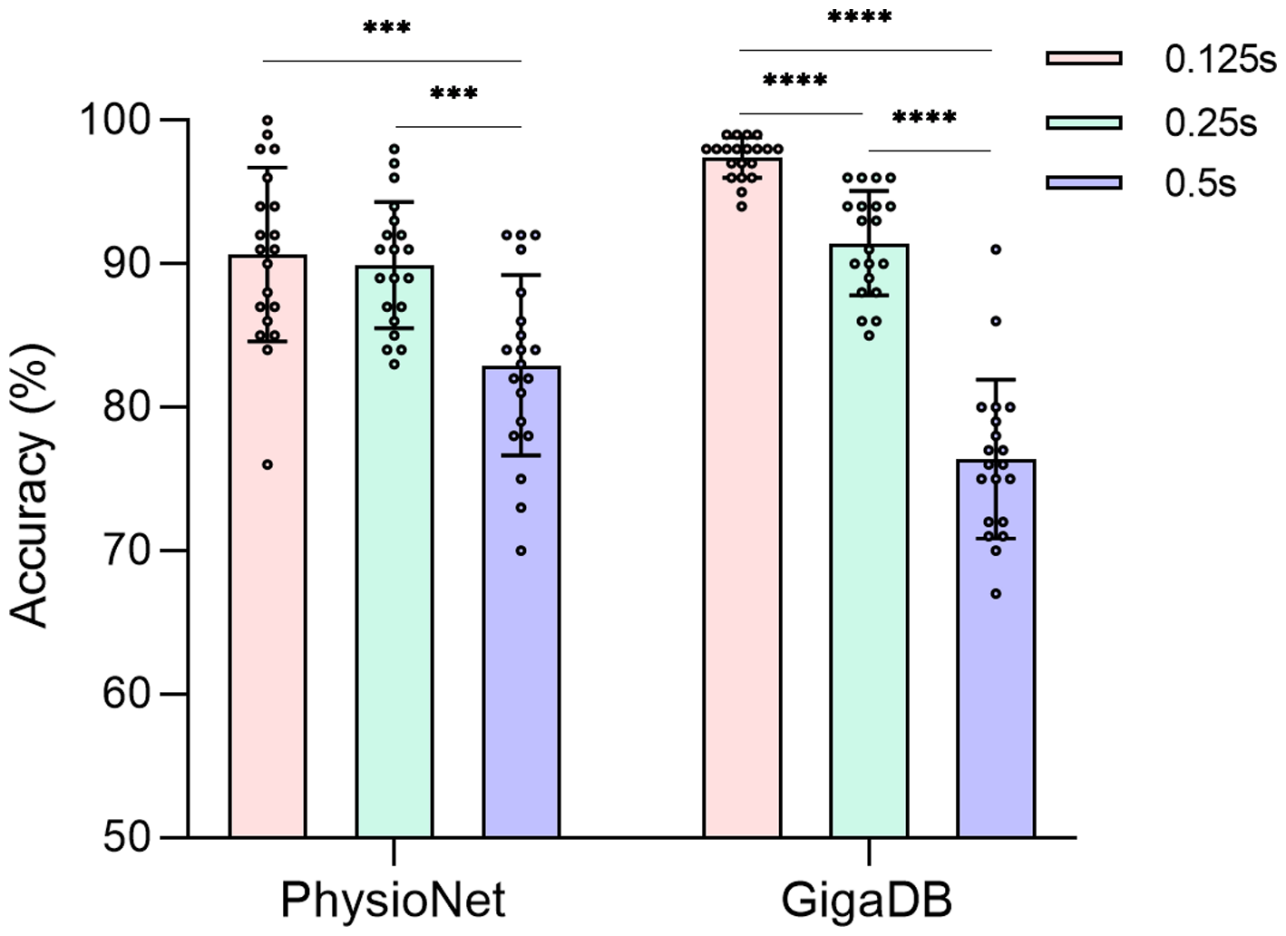
The accuracy of different sliding window steps in two datasets. The solid circle represents the classification accuracy of each subject. ***: *p* < 0.001. ****: *p* < 0.0001.

In order to evaluate the performance of the proposed 3D-CNN on two datasets (PhysioNet and GigaDB), we conducted the following ablative experiments. We investigated the impact of the sliding window strategy on the dynamic aspect of EEG signals and compared three different sliding window steps based on the proposed 3D-CNN. For each subject, considering the subject’s mental concentration, the length of the EEG signal in a single trial was fixed at 2 s. Smaller time steps in the sliding window strategy indicated greater dynamic variability, which means a higher degree of data augmentation for EEG. The sliding window steps were measured in terms of time steps. Accuracy was used to evaluate the classification results in the task. To ensure the reliability of the results, 20 accuracies was obtained by each subject and eliminating randomization effects. One-way ANOVA analysis was utilized to determine the presence of significant differences. As shown in Figure 5, we observed a gradual increase in classification accuracy as the time step decreased from 0.5 s to 0.125 s. Although the 3D-CNN achieved optimal performance on both datasets when the step size was set to 0.125 s (PhysioNet: *p* < 0.001, GigaDB: *p* < 0.0001), there was no significant statistical difference between 0.25 s and 0.125 s on the PhysioNet dataset. Moreover, both strategies yielded an average accuracy of over 90% on the GigaDB dataset. Taking into consideration computational complexity, dynamic variability and the balance between the two strategies across the datasets, we selected a sliding window step size of 0.25 s for subsequent experiments.

#### 3.1.2. Classification with subject-independent

**Figure 6:**
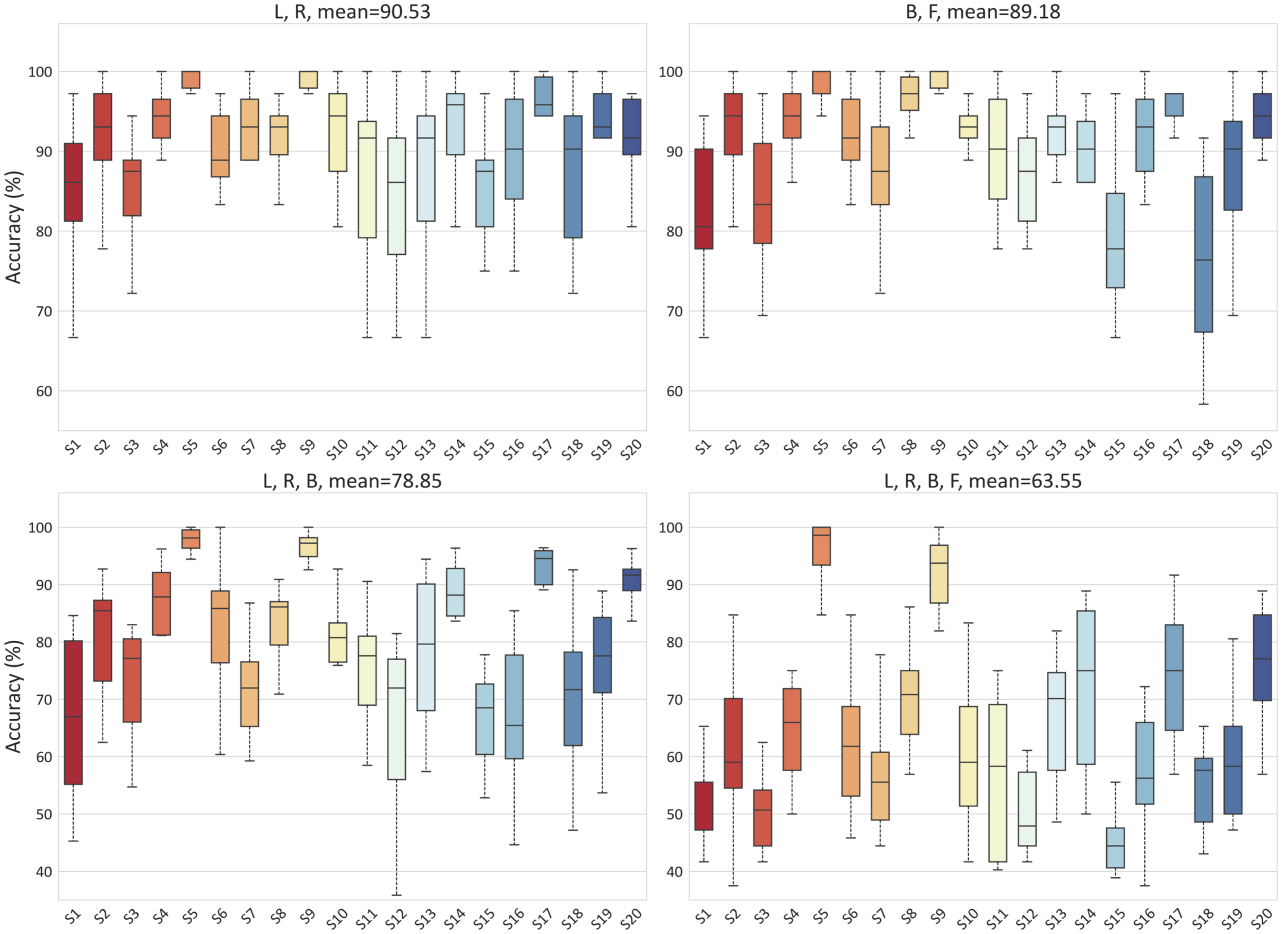
Classification accuracy of different categories in the PhysioNet dataset for MI.

**Figure 7:**
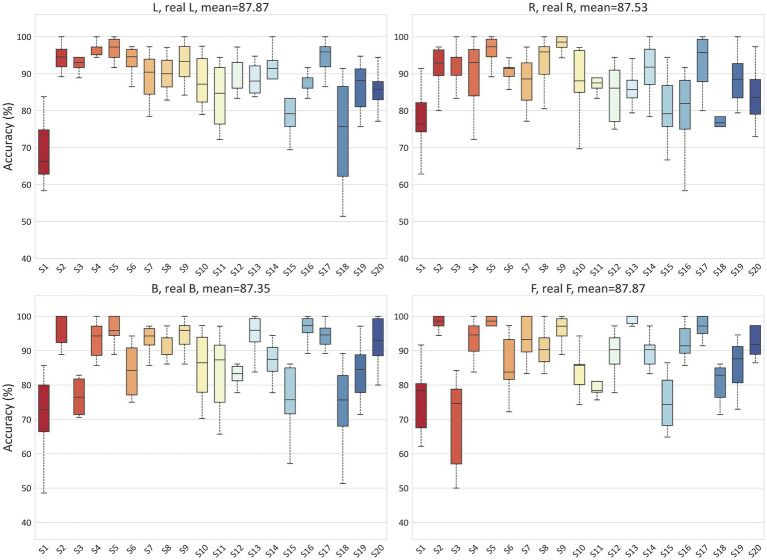
Classification accuracy of MI vs. ME in the PhysioNet dataset.

To validate the ability of the proposed model to handle EEG individual variability in both MI and ME scenarios, we conducted classification experiments on real and imagined movements. Due to inter-individual variability in EEG-based applications, we tested the accuracy for each subject separately, with each result being tested ten times. For the MI scenario, Figure 6 illustrates the classification results for 2-class (L, R), 3-class (L, R, B), and 4-class (L, R, B, F) in the PhysioNet dataset. On average, the accuracy for the L, R tasks, B, F tasks, L, R, B tasks, and L, R, B, F tasks in the PhysioNet dataset were 90.53, 89.18, 78.85, and 63.55%, respectively. For the MI vs. ME scenario, Figure 7 presents the average accuracy for L, real L tasks, R, real R tasks, B, real B tasks, and F, real F tasks in the PhysioNet dataset, which were 87.87, 87.53, 87.35, and 87.87%, respectively.

**Figure 8:**
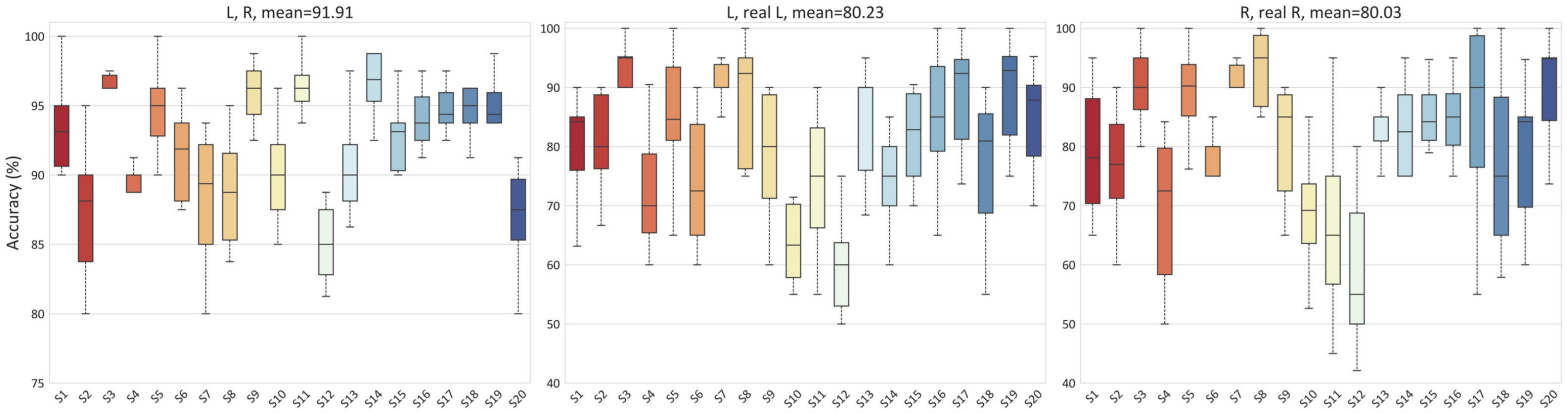
Classification accuracy of MI and MI vs. ME in the GigaDB dataset.

To demonstrate that our proposed method also achieves good performance on other datasets, we utilized the GigaDB dataset as additional data support. Figure 8 presents the average accuracy for the L, R tasks, L, real L tasks, and R, real R tasks in the GigaDB dataset, which were 91.91, 80.23, and 80.03% respectively, indicating that our model can achieve good performance in both the PhysioNet dataset and the GigaDB dataset.

### 3.2. Brain connectivity analysis

Based on the aforementioned experimental results, we can observe the best-performing subject (highest accuracy) and the worst-performing subject (lowest accuracy). To analyze the underlying reasons for these discrepancies, we can investigate from the perspective of brain functional connectivity. By examining the brain functional connectivity patterns of individuals, we can gain insights into the neural mechanisms associated with the observed variations in classification performance. Analyzing connectivity measures such as Pearson Correlation Coefficient (PCC), Coherence (COH), Phase Locking Value (PLV) can provide valuable information about the efficiency and coordination of brain regions involved in motor imagery tasks.

**Figure 9:**
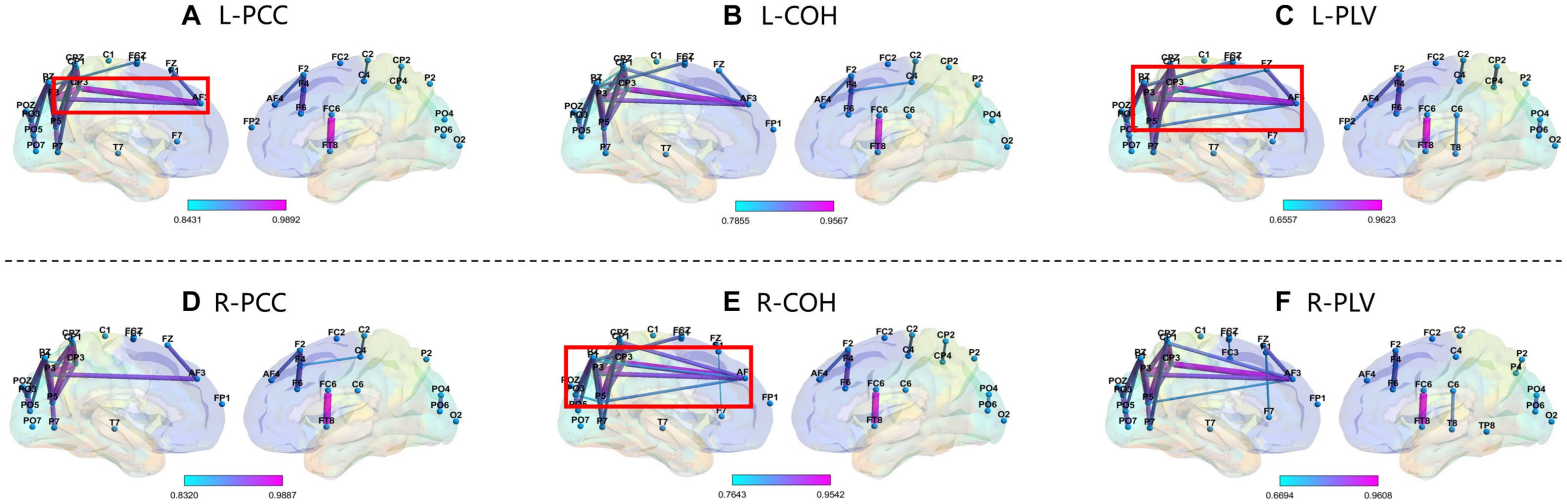
Top line: network topology of PCC, COH, and PLV for MI left-hand (L). Bottom line: network topology of PCC, COH, and PLV for MI right-hand (R). Please note that the data in this figure comes from S14. From top bold **(A–F)** are the part labels of the sub-figures.

**Figure 10:**
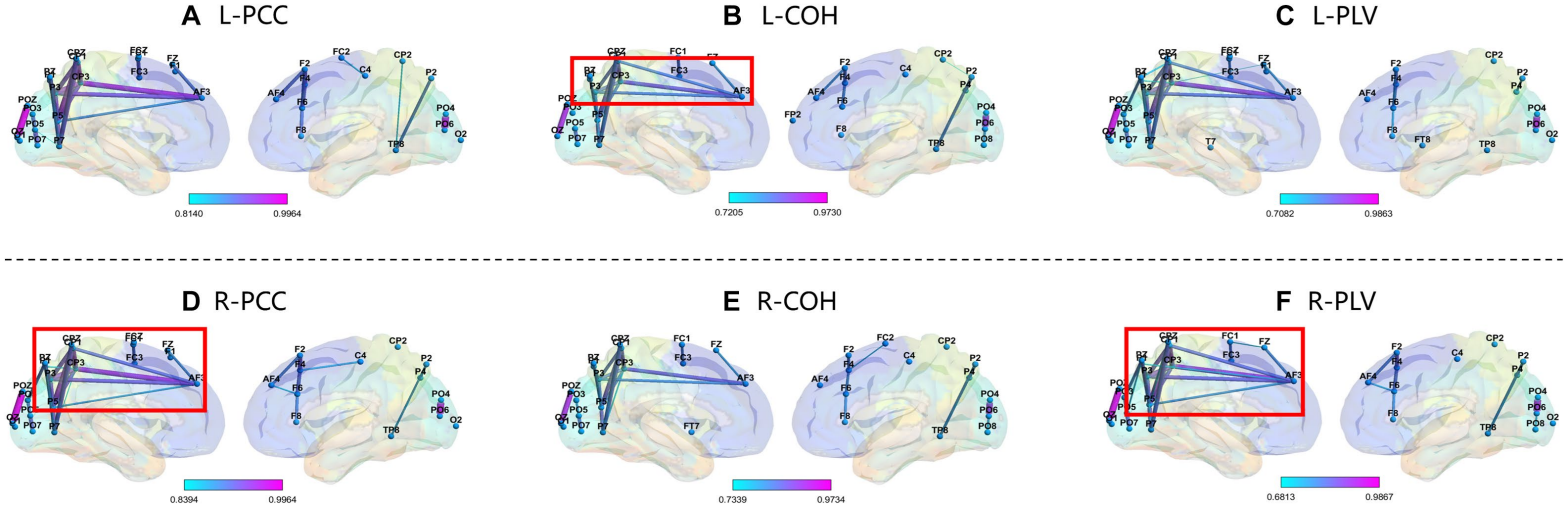
Top line: Network Topology of PCC, COH, and PLV for MI left-hand (L). Bottom line: Network Topology of PCC, COH, and PLV for MI right-hand (R). Please note that the data in this figure comes from S12. From top bold **(A–F)** are the part labels of the sub-figures.

We selected subject S14 (obtained the highest accuracy) and S12 (obtained the lowest accuracy) from the GigaDB dataset for brain network analysis. For the subject S14, the corresponding topological networks for two imaginary movements are shown in Figure 9. The thickness and color of the lines in the figures represent the strength of connectivity between different brain regions. These regions exhibit significant changes in functional connectivity under the influence of specific tasks. For better clarity, please refer to the figure where the red border is highlighted. As shown in Figure 9, it can be observed that during the MI left-hand and right-hand, there is an enhancement in the connectivity strength between brain regions. Specifically, there is an increase in the connectivity strength between the pre-motor area in the frontal lobe, the somatosensory association area in the parietal lobe, the visual association area in the occipital lobe, and the visual cortex. Upon further observation, it is exciting to note the presence of “triangular structures” and “circular structures” formed between the frontal lobe, parietal lobe, and occipital lobe within the highlighted red border. These structures are crucial features in complex networks, and a higher number of structural characteristics in brain connectivity indicates a more stable “communication” between brain regions. The “triangular structures” and “circular structures” highlighted in Figures 9A,C,E from the three networks (PCC, COH, PLV) complement each other in terms of revealing structural feature information, enabling a deeper exploration of EEG characteristics. As shown in Figure 10, we analyzed the network structural features corresponding to motor imagery of the left-hand and right-hand using data from subject S12. It was observed that although “triangular structures” and “circular structures” were also formed between different brain regions, the number of structures and connectivity strength were weaker compared to the functional connectivity of subject S14. This finding provides an explanation for the lower classification accuracy observed in subject S12. Furthermore, it suggests that the weaker network structural characteristics in S12’s brain may contribute to the reduced performance in classification accuracy.

The experimental results demonstrate the strengths and weaknesses of each method. By combining multiple approaches, more comprehensive and accurate analysis results can be obtained, taking advantage of the complementary nature of the three methods. This is also the reason why we combined the feature information generated by these three methods to form 3D features and designed a 3D-CNN for classification purposes.

## 4. Discussion

In this study, we have demonstrated the effectiveness of using the sliding window strategy to combine PCC, COH, and PLV features across different time segments as Temporal-Frequency-Phase Features (TFPF) for decoding EEG signals. In the classification and recognition process, we utilized a 3D Convolutional Neural Network (3D-CNN) model to extract and classify the 3D-TFPF representations. This approach improves classification performance and provides insights into the multi-domain feature information of EEG signals.

To elucidate these findings, we conducted classification experiments on MI and ME movements using two datasets. It is important to note that each experiment involved a single subject to assess the generalization capability of the proposed model across different individuals. Specifically, we analyzed the preprocessed EEG signals for each time segment to extract the PCC, COH, and PLV features, which were then stacked together to form a 3D feature representation. This method effectively captures the temporal, frequency, and phase-related information of the EEG signals while reducing their complexity. Furthermore, we employed a compact 3D-CNN model with two convolutional layers to classify 2-class, 3-class, and 4-class tasks based on the TFPF representations. The experimental results, as shown in Table 1, demonstrate that our proposed method outperforms other research approaches. These results highlight the improved performance of the proposed method in MI/ME classification and its ability to handle inter-individual variability challenges (Subject-Level).

**Table 1:** Comparison with state-of-the-art methods on the PhysioNet and GigaDB dataset.

Method	Subjects	Classification Type	Level	Avg. ACC	Dataset
DWT + DNN (Tolic and Jovic, 2013)	4	MI 2-class	Subject	68.21%	PhysioNet
Phase information (Loboda et al., 2014)	103	Real 2-class	Group	78.95%	
		MI 2-class	Group	71.55%	
SUTCCSP (Park et al., 2014)	56	MI 2-class	Group	72.37%	
IMOCS (Handiru and Prasad, 2016)	85	MI 2-class	Group	63%	
	35	MI 2-class	Group	79.90%	
MEMD + SUTCCSP (Kim et al., 2016)	24	MI 2-class	Group	80.05%	
CNNs (Dose et al., 2018)	105	MI 2-class	Group	87.98%	
		MI 3-class	Group	76.61%	
		MI 4-class	Group	65.73%	
G-CRAM (Zhang et al., 2020)	10	MI 2-class	Subject	74.71%	
**Proposed method**	**20**	**MI 2-class**	**Subject**	**89.86%**	
		**MI vs. ME 2-class**		**87.66%**	
		**MI 3-class**		**78.85%**	
		**MI 4-class**		**63.55%**	
OPTICAL (Kumar et al., 2019)	52	MI 2-class	Subject	68.19%	GigaDB
EEGG (Liu and Wang, 2022)	26	MI 2-class	Subject	78.09%	
**Proposed method**	**20**	**MI 2-class**	**Subject**	**91.91%**	
		**MI vs. ME 2-class**		**80.13%**	

*Level*: Using a group of subjects for testing or individual subjects for testing. *Classification Type*: Multiple classification types of motor imagery (MI) or real execution (ME). *Avg. ACC*: average classification accuracy based on test data. Note that MI 2-class on the PhysioNet corresponds to L vs. R, B vs. F tasks, L vs. real L, R vs. real R, B vs. real B, F vs. real F. MI 3-class corresponds to L vs. R vs. B. MI 4-class corresponds to L vs. R vs. B vs. F. While on the GigaDB, MI 2-class corresponds to L vs. R, L vs. real L, and R vs. real R. The bold text means the good performance of the proposed method under different MI/ME tasks.

Furthermore, compared to other existing methods, the combination of PCC, COH, and PLV offers several advantages. Firstly, it captures different aspects of brain connectivity, including linear correlations, frequency-specific coherence, and phase synchrony. This comprehensive analysis enhances our understanding of brain network dynamics. Secondly, the combination of these methods enables a more robust and reliable extraction of EEG features by considering both temporal, frequency and phase domains. Lastly, the integration of multiple methods provides a more holistic perspective on brain activity, improving the accuracy and interpretability of EEG analysis. Additionally, the utilization of multiple features overcomes the limitations associated with using a single feature type, enabling us to encapsulate the multifaceted and intricate dynamics inherent in EEG data. As shown in Figures 9, 10, identifying the factors that contribute to the varying performance levels among subjects can lead to a deeper understanding of individual differences in cognitive processes and brain dynamics. Moreover, such insights may guide the development of personalized approaches and interventions to enhance classification accuracy and improve the overall effectiveness of EEG-based MI systems.

Our research has certain limitations that should be taken into consideration when interpreting the study results. Firstly, the study primarily focused on offline analysis, and the proposed method has not been evaluated in real-time scenarios. Real-time implementation is essential for practical applications, and further research is necessary to explore the feasibility and performance of the method in real-time settings. Secondly, the performance of the proposed method is heavily dependent on the preprocessing techniques applied to the EEG signals. The choice of preprocessing techniques can have an impact on the results, including filtering, artifact removal, and feature extraction. It would be beneficial to further investigate and evaluate different preprocessing approaches to gain a better understanding of their influence on classification accuracy. Additionally, the study was conducted on relatively small-scale datasets, and it remains uncertain how the proposed method would perform when applied to larger datasets with more subjects and complex experimental designs. Assessing the scalability and efficiency of the method in handling larger datasets is crucial to determine its practical utility in real-world scenarios.

## 5. Conclusion

In conclusion, our study demonstrates the effectiveness of combining PCC, COH, and PLV features as Temporal-Frequency-Phase Features (TFPF) for decoding EEG signals. The utilization of a 3D Convolutional Neural Network (3D-CNN) model improves classification performance and provides insights into the multi-domain feature information of EEG signals. Furthermore, our findings contribute to the field of brain network analysis by enhancing our understanding of brain connectivity and dynamics.

Future work should focus on several aspects. Firstly, to ensure the practical applicability of the proposed method in real-world settings, it is imperative to conduct thorough evaluations of its performance and feasibility in real-time scenarios. We anticipate that the combination of the MI/ME real-world system with the multi-domain dynamic feature extraction method will generate considerable interest and attention within the Brain-Computer Interface (BCI) community. Another direction involves exploring multi-domain features in other EEG-based brain-computer interface (BCI) domains, such as emotion recognition and simulated driving classification. The proposed Temporal-Frequency-Phase (TFPF) representations, due to their generation scheme, are advantageous in preserving task-related multi-domain dynamics and can be readily applied to other BCI fields.

## Data availability statement

The original contributions presented in the study are included in the article/supplementary material, further inquiries can be directed to the corresponding authors.

## Author contributions

CF designed the experiment, carried out the overall design, and wrote the manuscript. BY provided manuscript ideas and 3D-CNN framework for writing. XL provided brain network analysis and data validation. PZ refined the ideas and provided the manuscript revision. All authors contributed to the article and approved the submitted version.

## Funding

This work was supported by the National Key Research and Development Program of China (2022YFC3602700 and 2022YFC3602703), National Defense Basic Scientific Research Program of China (Defense Industrial Technology Development Program) (Grant No. JCKY2021413B005) Shanghai Major science and technology Project (No. 2021SHZDZX), the Shanghai Industrial Collaborative Technology Innovation Project (No. XTCX-KJ-2022-2-14).

## Conflict of interest

The authors declare that the research was conducted in the absence of any commercial or financial relationships that could be construed as a potential conflict of interest.

## Publisher’s note

All claims expressed in this article are solely those of the authors and do not necessarily represent those of their affiliated organizations, or those of the publisher, the editors and the reviewers. Any product that may be evaluated in this article, or claim that may be made by its manufacturer, is not guaranteed or endorsed by the publisher.

## Footnotes

^1^Publicly available at https://archive.physionet.org/pn4/eegmmidb/.

^2^Publicly available at http://gigadb.org/dataset/100295.
